# Evaluation of Photocatalytic, Antioxidant, and Antibacterial Efficacy of Almond Oil Capped Zinc Oxide Nanoparticles

**DOI:** 10.3390/ma16145011

**Published:** 2023-07-15

**Authors:** Iqra Ramzan, Mahwish Bashir, Adnan Saeed, Babar Shahzad Khan, Mohammed Rafi Shaik, Merajuddin Khan, Baji Shaik, Mujeeb Khan

**Affiliations:** 1 Department of Physics, Government College Women University, Sialkot 51310, Pakistan; ramzaniqra9@gmail.com (I.R.); mahwish.bashir@gcwus.edu.pk (M.B.); adnan.saeed@gcwus.edu.pk (A.S.); 2 Department of Chemistry, College of Science, King Saud University, P.O. Box. 2455, Riyadh 11451, Saudi Arabia; mrshaik@ksu.edu.sa (M.R.S.); mkhan3@ksu.edu.sa (M.K.); 3 School of Chemical Engineering, Yeungnam University, Gyeongsan 38541, Republic of Korea; shaikbaji@yu.ac.kr

**Keywords:** almond oil, ZnO, solvothermal, molar ratio, photocatalytic, antibacterial, antioxidant

## Abstract

In this study, ZnO nanoparticles (NPs) were synthesized in the presence of almond oil at various molar ratios of zinc acetate and sodium hydroxide, including 0.5:1, 0.75:1, 1:1, 1.25:1, and 1.5:1, to obtain pH values of 11, 10, 9, 8, and 7, respectively. The XRD results revealed that ZnO NPs exhibit a hexagonal structure, with high crystallinity. SEM results showed that dense and large sized ZnO NPs were formed at pH 11, and relatively small (~30–40 nm) NPs were obtained at pH 9. The size distribution can be explained in terms of the presence of $OH^{-}$ ions at different pH levels. However, the larger size of the NPs at pH 7 compared to those at pH 8–11 were due to the coalescence of NPs suitable for antioxidant/antibacterial activities. ZnO NPs demonstrated a high degradation efficiency (~93%) in 90 min, with a high rate constant for Methyl Orange (MO), which is better than the previously reported rate. The larger sized almond oil capped ZnO NPs also showed excellent radical scavenging activity (94%) and are proven to be good carriers to resist *Escherichia coli* (*E. coli*) bacteria.

## 1. Introduction

Rapid industrial development has adversely increased environmental pollution, such as air and water contamination, which ultimately causes several ecological issues. Various industries release a significant amount of toxic and hazardous pollutants into the environment, the presence of which even minute concentrations is a serious threat [1,2]. Among various pollutants, dyes generate the most severe water pollution and can have dangerous impacts on living organisms. Therefore, the effective removal of these contaminants from water is highly desired [3,4].

The conventional methods such as physical adsorption, biodegradation, and coagulation partially degrade dyes and produce secondary hazardous pollutants in their solid forms [5,6,7]. In contrast, semiconductor photocatalytic technology could be adopted as an effective alternative approach for the removal of these hazardous dyes using solar energy [8,9]. The application of light on the photocatalytic oxide generates electron hole pairs which convert the adsorbed H_2_O and O_2_ molecules into reactive oxygen species, such as hydroxyl radical (OH) and superoxide anions (O_2_^−^). The produced reactive oxygen species degrade persistent organic pollutants (POPs) and reduce heavy metal pollutants [10,11]. A variety of semiconductor oxide-based photocatalysts such as ZnO [12], In_2_O_3_ [13], TiO_2_ [14], CuO [15], etc., have been extensively studied for water purification.

Among these photocatalysts, ZnO is recognized as the most promising potential candidate for the photodegradation of dyes such as Methyl blue (MB), Methyl orange (MO), Prussian blue, etc. ZnO has a bandgap (binding energy) of 3.37 eV (60 meV) and is capable of absorbing ultraviolet radiation [16]. Due to its extraordinary physiochemical, electronic, and antibacterial characteristics, it is used in various electronic and optoelectronic devices, such as transistors, biosensors, catalysts/photocatalysts, ultraviolet absorbers, pigments, photoelectrodes, solar cells, antibacterial coatings, etc. [17,18].

The literature reports many methods used to synthesize photocatalytically active ZnO NPs, including thermal evaporation, CVD [19], PVD, mechano-chemical preparation [20], the sol–gel process [21], spray pyrolysis [22], precipitation [23], etc. These methods are relatively expensive and produce low yields. In contrast, the chemical approach is a simple, economically viable, environmentally friendly, and high yield synthesis technique. Additionally, synthesized nanomaterials exhibit high purity, as well as various morphologies which could be controlled by the optimization of the reaction conditions [6].

However, the preparation of stable and non-agglomerated ZnO NPs is a challenging task. The agglomeration of particles takes place due to their magnetic nature, which has a detrimental effect on applications as it reduces the effective bulk surface area [24]. The inter-particle interactions caused by Van der Waals forces and dipole–dipole interactions are mainly responsible for the agglomeration of nanoparticles [25,26]. Capping agents can be used in NPs formulation for increased stability while preventing undesired changes, such as aggregation. These agents should be able to interact with metal ion and subsequently prevent aggregation through steric hindrance. The surface chemistry and size distribution of nanoparticles become altered after being capped with biocompatible surfactants [27,28,29,30]. Capping agents should be biodegradable, well-dispersed, biosoluble, biocompatible, and non-toxic in nature so that they can be easily utilized in living systems. Hence, their non-specific interaction with biological components is reduced, leading to alleviated cellular toxicity [31]. The frequent use of capping agents in colloidal dispersions is employed to regulate nanoparticles, controlling the growth, agglomeration, and physicochemical characteristics in a precise way [32].

Particularly, the functionalization of nanomaterials using vegetable oils is considered as effective technique [33]; still, these materials have been relatively less utilized. Among various vegetable oils, almond oil, which is widely extracted for various purposes including for use in food flavorings and the cosmetics industry [34], is a rich source of macronutrients and micronutrients. Almond oil is comprised of a large proportion of poly and monounsaturated fatty acids, including oleic acid as the main component, which has been effectively utilized as capping agent for a variety of nanoparticles [35]. Thus, almond oil, with a large oleic acid content, can be a potential functionalization agent, possibly facilitating the formation of functionalized nanoparticles for various applications [36]. Almond oil may acts a capping agent, potentially reducing the agglomeration of NPs and altering the surface properties of nanomaterials to produce functional NPs, including ZnO [34]. In the literature, almond gum and almond shells were used in the synthesis of ZnO NPs [37,38], but to our knowledge, this study marks the first time that almond oil has been used as a capping and stabilizing agent for ZnO NPs.

ZnO based materials have been efficiently used as photocatalyst for the photodegradation of hazardous organic dyes, including methyl orange (MO) [39]. Molaei et al. synthesized egg white/glycerol mediated ZnO nanorods and observed a nearly 90% degradation [40]. Kale et al. synthesized ZnO and W doped ZnO for photocatalytic applications and observed an 82.07% degradation against crystal violet in 5 wt% doped ZnO [41,42]. Flower-like ZnO nanostructures synthesized using the polyvinyl pyrrolidone (PVP)-assisted co-precipitation route demonstrated a 75% photo-degradation efficiency against MO in 240 min [43]. Spindle-like ZnO microparticles were synthesized by a simple hydrothermal method using triethanolamine (TEA) as a surfactant. These structures were reported to show a removal efficiency of 95% for MO in 180 min [44]. The aforementioned literature suggests that the synthesis of NPs involves surfactant mediated routes. However, the synthesis of ZnO nanostructures involving naturally available constituents with high photocatalytic activity against hazardous dyes is still highly desirable.

**Figure 1:**
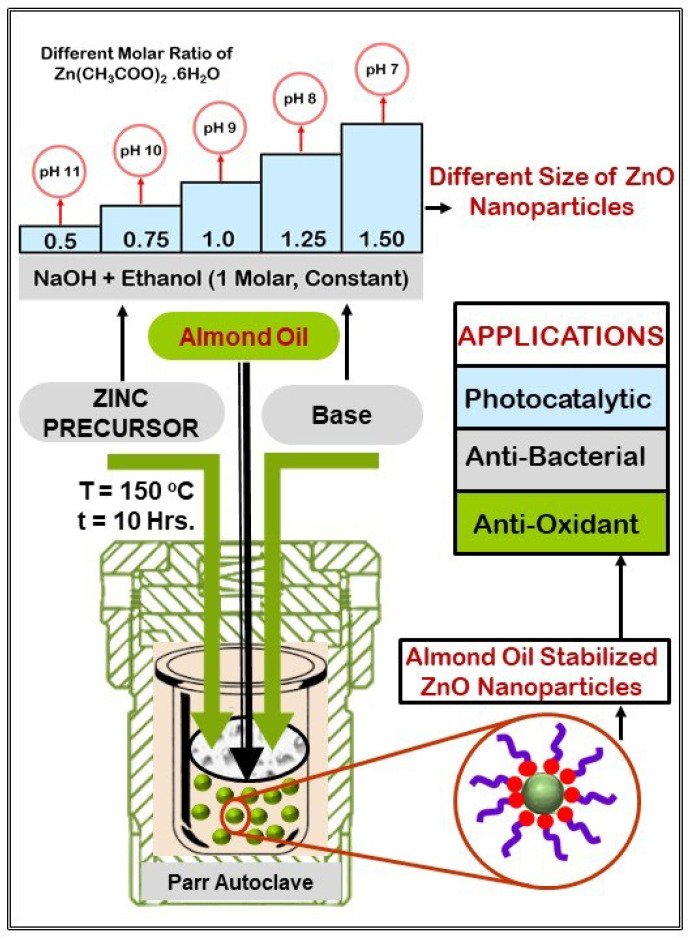
Schematic representation of experimental parameters and applications of almond oil stabilized ZnO NPs.

One of the important uses of ZnO NPs is for antibacterial activity, which refers to the destruction of bacteria and/or the obstruction of their growth without causing harm to cells and tissues. Bacillus subtilis, *Escherichia coli* (*E. Coli*), *Staphylococcus Aureus* (*S. aureus*), and other microbial contaminants need to be effectively and efficiently eliminated because they pose a serious threat to the environment, industry, healthcare, food, bones, and other areas [45]. The Gram-negative rod-shaped bacterium *E. coli* is the most significant rotting bacteria causing bread, meat, and dairy products to spoil [46,47,48]. Human intestines are home to the *E. coli* bacteria, which can occasionally result in kidney failure and cause diarrhea, vomiting, cramps, and stomach pain. In regards to the antibacterial activity of ZnO NPs against the aforementioned bacteria, numerous studies have been reported [49]. Therefore, in the present work, almond oil capped ZnO NPs were prepared using an eco-friendly solvothermal route. Moreover, different sizes of ZnO NPs were prepared by adjusting the pH of the reaction media using varied ratios of precursor to base. The resulting stabilized ZnO NPs were characterized by using different spectroscopic and microscopic techniques. Moreover, the as-prepared almond oil capped ZnO NPs were applied as effective photocatalyst for the degradation of MO hazardous organic dyes. In addition, ZnO NPs were also utilized an anti-bacterial agents towards the inhibition of different pathogens (cf. Figure 1). 

## 2. Materials and Methods

Zinc Acetate hexahydrate (Zn(CH_3_COO)_2_.6H_2_O) (Sigma-Aldrich, St. Louis, MO, USA), without purification, was used as a precursor of ZnO. Precursors play an important role for nanostructure morphology. Chlorides/acetates cause surface adsorption and form spherical particles, while nitrates form non-coordinative ligands and preferably, c-axis growth occurs. As compared to chlorides, acetates offer a spherical morphology due to a rapid decomposition process [50]. Virgin almond oil is used as the capping agent.

### 2.1. Extraction of Almond Oil

Prior to extracting almond oil, the pericarp and epicarp were removed. Then nuts were dried in an oven and then pressed using a hydraulic press. This process involves cold methods, which do not disturb the characteristics of the almonds. Moreover, this system is cost-effective and does not involves hazardous chemical substances [34]. 

### 2.2. Synthesis of ZnO NPs

Zinc acetate hexahydrate (Zn(CH_3_COO)_2_.6H_2_O) was mixed in ethanol under constant magnetic stirring at 60 °C, and the solution exhibited a pH of ~6.5. Another solution was prepared by mixing NaOH in ethanol. To obtain the molar ratio of Zn(CH_3_COO)_2_.6H_2_O:NaOH of 0.5:1, the NaOH solution was added dropwise under vigorous stirring. Upon the addition of NaOH, the pH of the resulting solution varied. After the adjustment of pH, 1 mL of extracted almond oil was added, and the mixture was stirred for 2 h. This mixture was transferred into autoclaves and maintained at 150 °C for 10 h under autogenous pressure. After the reaction was complete, the resulting white solid products were washed three times with ethanol and DI water. The final product was dried in a furnace at 60 °C for 1 h. These synthesized NPs were washed with distilled water and ethanol, centrifuged at 3000 rpm for isolation, and then dried at 50 °C for powder formation. Similarly, five different samples of ZnO were prepared by varying the molar ratios of Zn(CH_3_COO)_2_:NaOH, using the same procedure.

Herein, to vary the pH of the solution, a fixed concentration of NaOH was maintained, while the amount of the precursor was increased in the solution, which in turn varied the pH of the solution from 11 to 7. Five different molar ratios of $\mathrm{Zn}{\left({\mathrm{CH}}_{3}\mathrm{COO}\right)}_{2}.6H_{2}O:\mathrm{NaOH}$ were maintained, including 0.5:1, 0.75:1, 1:1, 1.25:1, and 1.5:1, which led to different pH values of 11, 10, 9, 8, and 7, respectively. The crystallization and size of the ZnO NPs increased due to a higher pH, and the high pH also led to the formation of spherical shaped ZnO NPs, while nanorods/nano flowers were formed at an acidic pH, results which are also reported in the literature [51,52]. Various researchers [53,54,55] have previously synthesized ZnO NPs, varying the molar ratio of zinc acetate and sodium hydroxide ratios without a surfactant. Dejene et al. [56] synthesized ZnO nanoparticles by varying molar ratios of zinc acetate and NaOH and obtained a pH ~5.6 to 11.5. They performed the synthesis without a capping agent; therefore, the presence of a high degree of agglomeration and random size distribution was observed. Similar observations were obtained by Osman and Mustafa [54] in the absence of a capping agent. According to the literature [57], the use of a capping agent is necessary to prevent agglomeration and enhance the efficiency for biological applications. Capping agents provide colloidal stability and prevent the random growth of NPs.

The experimental parameters and applications of solvothermally synthesized almond oil capped ZnO NPs are presented in Figure 1.

### 2.3. Characterizations

Phase identification of the samples was performed using a Bruker D8 advance X-ray diffractometer with *CuKα* (λ = 0.15406 nm), a scanning range of 20–80 degrees, and a step size of 0.05. The crystallite size was calculated by employing the Scherrer equation (Equation (1)) [58].

(1) $$D=\frac{k\lambda }{Bcos\theta }$$

where *D* = crystallite size, *K* = constant, and *λ* = 1.5406 Å. The number of dislocations per unit area, which are determined by the relation given in Equation (2), is known as the dislocation density [59].

(2) $$\delta =\frac{1}{D^{2}}$$

The lattice parameters of almond oil capped ZnO NPs are calculated using Equation (3) [58].

(3) $$\frac{1}{d_{hkl}^{2}}=\frac{4}{3}\left(\frac{h^{2}+k^{2}+l^{2}}{a^{2}}\right)+\frac{l^{2}}{c^{2}}\;$$

The scanning electron microscope TESCAN model MIRA3 was used to obtain the morphology of the prepared samples, with a 20 kV operating voltage. Fourier transform infrared spectroscopy (FTIR Bruker Tensor II) was used to examine the functional group and bond analysis of the almond oil capped ZnO NPs. The ZnO bands moved slightly toward lower wavenumbers, with variations in the pH/molar ratio. This can be explained by Equation (4) [60].

(4) $$ʋ=\frac{1}{2\pi c}\frac{\sqrt{K}}{\sqrt{\mu }}$$

where *c* = speed of light, *µ* = reduced mass, and *k* = force constant.

The UV-Vis spectra was obtained using the SPECORD 210 PLUS spectrometer. The band-gap calculated from Equation (5) is given below:

(5) $$E_{g}=\frac{1240}{\lambda }$$

#### Photocatalytic Activity

The evaluation of photocatalytic activity was conducted under visible light (200 W xenon lamp, *λ* > 420 nm) using MO dye. First, the photocatalyst amounts of 0.3, 0.6, and 0.9 g were mixed in 100 mL of pollutant solution. Then, the continuous magnetic stirring of the solution was performed for 20 min, maintaining the solution at a ~25 °C reaction temperature to attain the adsorption–desorption equilibrium. Finally, 3 mL of the prepared solution was separated and centrifuged. UV–Vis spectrophotometry (at 465 nm wavelength) was used to evaluate the concentration of MO. 

The degradation in the absorption was calculated using Equation (6) [61],

(6) $$Degradation\;rate\;\left(\%\right)=\frac{A_{0}-A}{A_{0}}$$

where $A_{0}$ is the initial dye absorption without UV irradiation, and *A* is the absorption after UV irradiation.

### 2.4. Antibacterial Study

To assess the extracted materials’ antibacterial effectiveness against Gram-negative bacteria *E. coli* (ATCC25922) and *Pseudomonas aeruginosa* (ATCC27853), the sample was sub-cultured on Lauria Bertani medium (LB) and incubated for 24 h at 37 °C. The disc diffusion method was used to assess the antibacterial activity. The studied strain was uniformly injected into the LB medium using a sterile swab and a saline suspension (NaCl 0.9%), which had previously been calibrated using the 0.5 McFarland standard. The culture substrate was covered with 6 mm diameter Whatman paper discs, which were subsequently dipped into the tested extract [62]. The inhibitory halos were incubated for 24 h at 37 °C, and their diameter was determined. 

### 2.5. Antioxidant Study

The ability of almond oil capped ZnO NPs prepared with a molar ratio of (1:5:1) to scavenge radicals was tested. The DPPH (1,1,diphenyl-2-picryl hydrazyl) assay was used to examine the antioxidant capabilities of the generated samples. The main mechanism underlying the antioxidant effect is the decolorization (purple to bright yellow) caused by the neutralization process. The suitable amount of NPs was added to the DPPH and methanol solution. For 30 min, the entire combination was stored in a dimly lighted, 37 °C chamber. The measurements were verified using data from the decolorization and the absorbance peak at 517 nm. The percentage of scavenging activity was calculated using Equation (7) [63];

(7) $$Free\;raddical\;scavenging\;activity\left(\%\right)=\frac{A_{Blank}-A_{Sample}}{A_{Blank}}\times 100$$

## 3. Results

### 3.1. XRD Analysis

**Figure 2:**
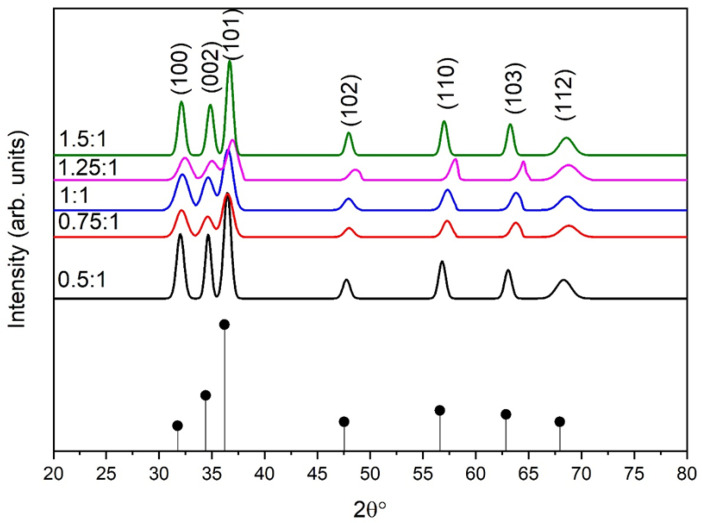
XRD of almond oil capped ZnO NPs prepared at different molar ratio of Zn(CH_3_COO)_2_.6H_2_O: NaOH.

The phase analysis of synthesized almond oil stabilized ZnO NPs at different molar ratios of Zn(CH_3_COO)_2_.6H_2_O:NaOH is shown in Figure 2. XRD analysis revealed the crystalline nature of ZnO NPs. Samples synthesized at all molar ratios of Zn(CH_3_COO)_2_.6H_2_O:NaOH showed constructive diffraction. The ZnO NPs exhibited a wurtzite hexagonal structure, which is confirmed by the reflection planes along (1 0 0), (0 0 2), (1 0 1), (1 0 2), (1 1 0), (1 0 3), (1 1 2), which are well matched with those on ICDD PDF card no 36-1451. 

The general observation of hexagonal peaks are noted when h + 2 k = 3 n with ‘I’ even, for example (002), and when h + 2 k = 3 n ± 1 with ‘I’ odd, for example (110), etc., as the observed d-spacing is similar to the hexagonal ZnO standard data, which ultimately matches the reflection planes [64]. No other peaks related to impurity were been observed in the samples. Samples prepared at the molar ratio of 0.5:1 show higher crystallinity than the other samples containing high concentrations of NaOH, except for the sample at a ratio of 1.5:1. The strong basic nature (pH 11) of the sample and the higher amount of OH ions facilitated the efficient nucleation and growth of ZnO NPs. More precisely, the basic nature of the sample speeds up the polycondensation phenomenon and results in a strong and non-agglomerated structure. The intensity of the peaks decreases as the pH of the samples reduces to 10, 9, and 8. However, for molar ratio 1.5:1 with pH 7, relatively high intensity peaks were again observed due to presence of more solute atoms. This entire phenomenon can be attributed to an increase in the electrostatic interaction among the atoms, resulting the increase in intensity and crystallinity of capped ZnO NPs.

### 3.2. Gaussian Fitting 

Data obtained from XRD analysis is further analyzed by Gaussian mathematical model fitting. For the sample prepared at molar ratio 0.5:1 (Figure S1a, Supplementary File), the hexagonal structure of ZnO observed with FWHM at 2θ = 36.489158° was 0.79003. As the molar ratio increases up to 0.75:1, the intensity of the peaks decreases due to reduction of OH ions (pH drops from 11 to 10), and FWHM increases are observed at a characteristic peak of 2θ = 36.5°, with FWHM = 1.08344 (Figure S1b). An additional increase in molar ratio or solute atoms decreases the pH of the solution, and a relatively higher FWHM 1.2229 and 1.20630 were observed for the sample with molar ratios 1:1 and 1.25:1, respectively (Figure S1c,d). In regards to molar ratio 0.5:1, FWHM increases, which confirms that crystallinity decreases. At molar ratio 1.5:1, FWHM again decreases to 0.74 due to the increased electrostatic interaction. Gaussian fitting is also applied to all peaks to determine Area Intg P. The area of characteristic peaks at molar ratios 0.5:1, 0.75:1, 1:1, 1.25:1, and 1.5:1 is 36.6%, 28.7, 30.5, 25.4, and 26.9%, respectively, showing that a decrease in pH reduces the crystallinity of the samples. A decrease in FWHM is observed at molar ratios up to 1.25:1, which is due to variation in Zn^2+^ and *OH*^−^ ions [25]. 

### 3.3. Crystallite Size and Dislocation Density

**Figure 3:**
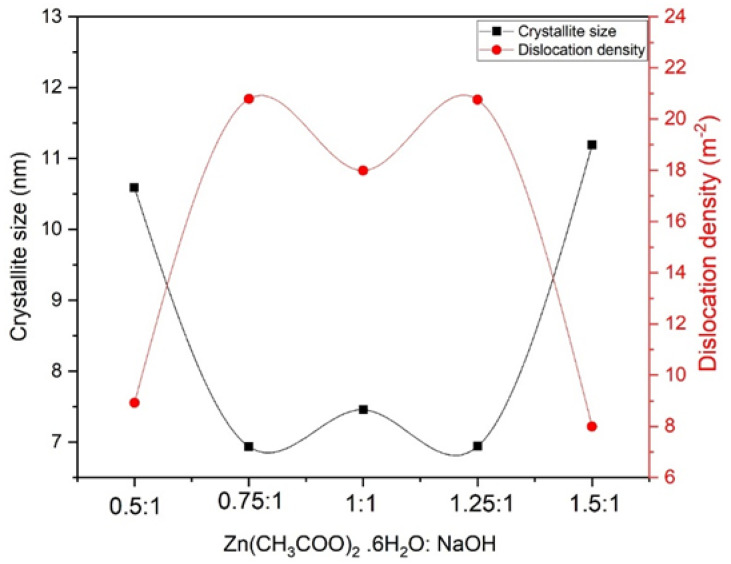
Crystallite size and dislocation density for different molar ratios of zinc acetate and sodium hydroxide.

Samples synthesized at molar ratio 0.5:1 showed higher crystallite size. Larger crystallite size is caused by the availability of more hydroxyl ions. These OH^−^ ions cause strong bonding and the emergence of NPs. Additional changes in molar ratio cause an abrupt decrease in crystallite size due to the minimum solubility of Zn(OH)_x_O_y_. On the other hand, the pH of the solution is varied by increasing the solute atoms. When the concentration of the starting solution is higher (1.5:1), more solute atoms are present, and more zinc cations will be bound together; thus, zinc ions come together to form clusters or agglomeration. It can be seen from Figure 3 that a relatively low dislocation density has been observed for samples having a larger crystallite size, which is due to the emergence and high growth rates of NPs. It is also observed that δ is low at molar ratio 1.5:1 due to increased crystallite size. Table 1 shows the calculations evaluated from XRD data [65]. The lattice parameters slightly decrease with an increase in molar ratios. An increase in lattice parameters has been observed with increased molar ratios, as a slight shifting towards higher angles in the peaks has been observed (Figure 2). 

### 3.4. Morphological Analysis

**Figure 4:**
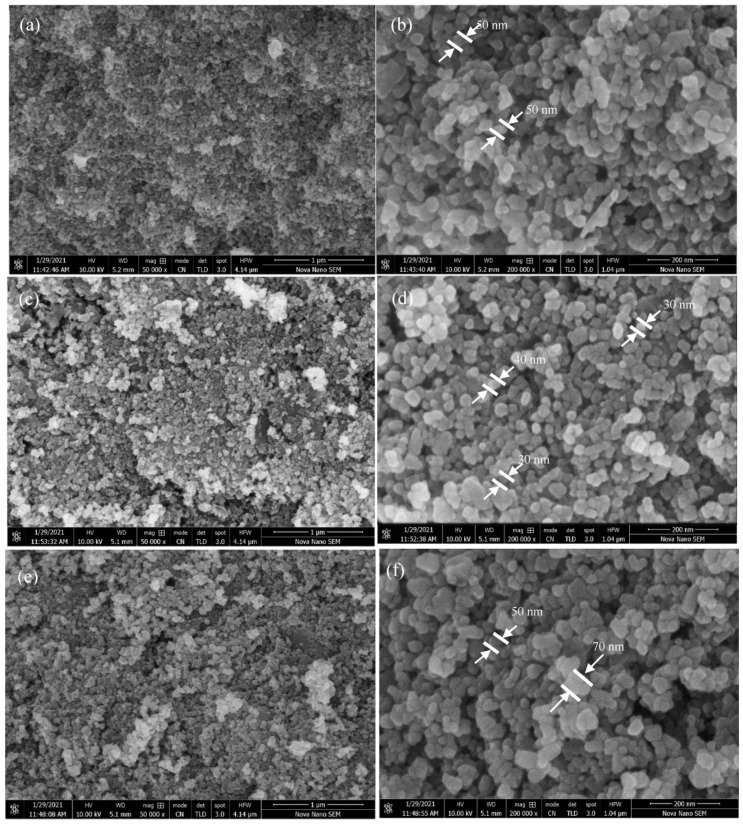
SEM images of hydrothermally synthesized ZnO at different molar ratios (**a**) 0.5:1, (**b**) magnified view at 0.5:1, (**c**) 1:1, (**d**) magnified view at 1:1, (**e**) 1.5:1, and (**f**) magnified view at 1.5:1.

Figure 4 shows the surface morphology of synthesized almond oil capped ZnO NPs, which demonstrate a sphere-like morphology of the resulting nanoparticles in all the samples. From the SEM images, it can be inferred that both at the highest pH (pH 11) and lowest pH (pH 7), slightly irregular shapes and relatively larger sized NPs were formed, while at the optimum pH (pH 9), spherical-shaped, smaller sized ZnO NPs were obtained. Although it is reported that at basic pH values, stable, homogeneous, and narrow sized distributed ZnO NPs are formed, even under basic conditions at different basic pH values (i.e., between the basic pH window of 7 to 11), the size of the resulting ZnO NPs may vary [66]. For example, the sample synthesized at molar ratio 0.5:1 (NaOH and precursor), which delivered the highest pH value (pH = 11), relatively non-uniform and larger sized NPs were formed (Figure 4a,b). Whereas, under reverse conditions, where the precursor is present in large amounts and the amount of the base remained the same, i.e., 1.5:1, a weak basic condition was obtained, where the pH was recorded as 7. Even in this condition, irregular shaped, larger sized ZnO NPs were formed (>50 nm). However, under optimum basic conditions, i.e., pH 9, which were achieved by using equivalent amounts of both the precursor and base (1:1), well-separated and well-defined spherical particles with relatively small sized NPs (30–40 nm) were formed, as shown in Figure 4d. Notably, in all the cases, between pH 7 to 11, less-agglomerated, different sizes of ZnO nanoparticles were formed due to the presence of almond oil as a capping agent. However, the size variation of the particles at different basic pH values can be attributed to the fact that at a higher pH (11), or in alkaline medium, hydrodynamic and crystallite size increased due to the high nucleation rate, which led to the formation of larger ZnO NPs (cf. Figure 4b). Whereas, under optimum conditions, consisting of an equivalent molar ratio of the precursor and base, the controlled number of solute atoms present in the solution and the balanced concentration of *OH*^−^ ions reduce the agglomeration and size of the nanoparticles. Therefore, for the sample prepared at molar ratio 1:1 (pH = 9), comparatively fewer *OH*^−^ ions present than in the sample obtained at pH 11, thus allowing the easy access of the solute ions to the nucleation sites, which resulted in the formation of smaller sized nanoparticles. However, at the lowest pH achieved under the molar ratio of 1.5:1, although the pH of the sample was low, the quantity of precursor ions increased, which resulted in the coalescence of NPs, thus leading to the formation of comparatively larger NPs (cf. Figure 4e,f), which was also indicated by the XRD data. This reveals that the concentration of the solute plays an important role in defining the size and morphology of the nanoparticles. For example, at a high molar ratio in the precursor (1.5:1), the number of Zn^2+^ ions increased in the solution, which caused the supersaturation, leading to the fast and uncontrolled nucleation and growth of the nanoparticles. This phenomenon can be explained by Gibbs free energy, which increases with the number of solute atoms in a solution. By separating the solute from the solution, the overall energy of solution is decreased [67]. These highly crystalline, stable, and spherical-shaped ZnO NPs are well suited for photocatalytic application.

### 3.5. EDX Spectra

**Figure 5:**
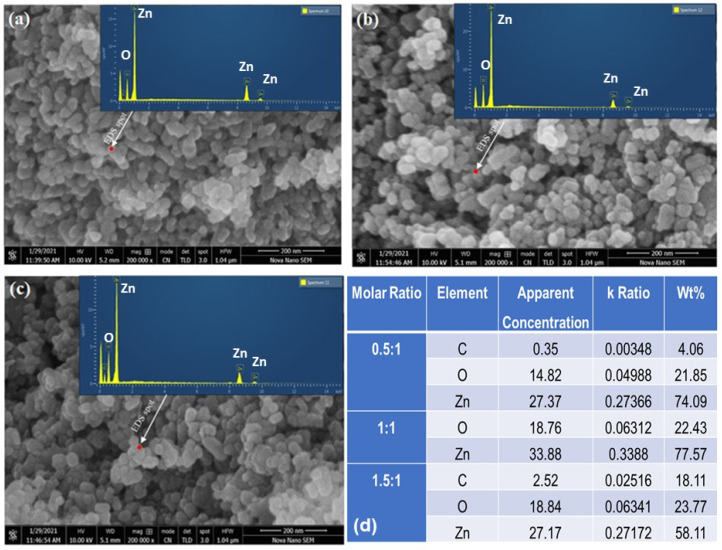
EDS location of samples prepared at molar ratios: (**a**) 0.5:1, (**b**) 1:1, and (**c**) 1.5:1; (**d**) composition characteristics of ZnO NPs prepared at different molar ratios.

**Figure 6:**
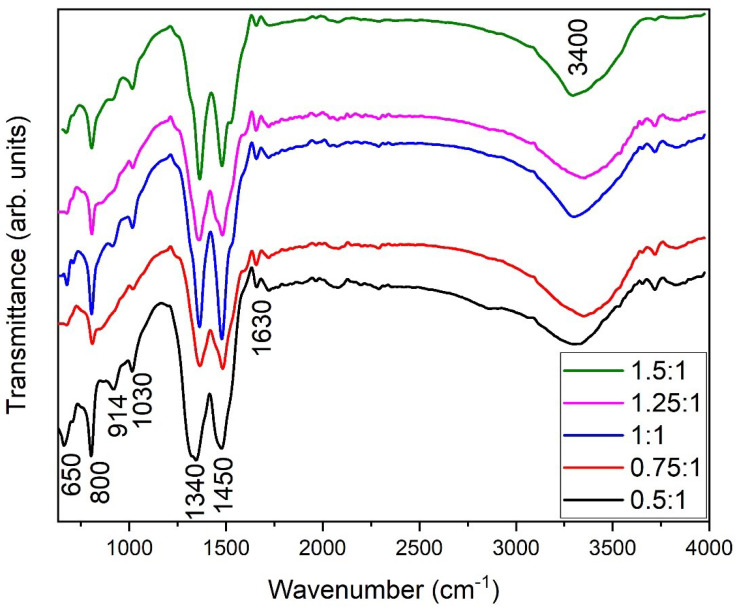
FTIR spectra of almond oil capped ZnO NPs prepared at different molar ratios.

According to the energy dispersive spectroscopy (EDS) of almond oil capped ZnO NPs prepared at molar ratios of 0.5:1 (Figure 5a), 1:1 (Figure 5b), and 1.5:1 (Figure 5c), the stoichiometry of the composition was obtained by selecting specific locations. In all the samples, the peaks of different elements, including carbon and oxygen in the EDS spectra occurred at ~0.25 and ~0.5 KeV, respectively, while the EDS peak of zinc appeared at different locations, i.e., ~1, ~8.7, and ~9.7 KeV. The presence of carbon peaks in all the spectra can be attributed to the stabilization of ZnO NPs by the almond oil, which contains oleic acid and other phytoconstituents. The EDS spectra revealed a strong signal for zinc at around ~74.09% and a prominent oxygen peak, with 21.85% for molar ratio 0.5:1, as described in Figure 6d. The sample prepared at molar ratio 1:1 shows a slight increase in zinc content up to 77.57% and an oxygen peak with a value 22.43%. This EDX result further confirms the synthesis of almond oil capped ZnO NPs.

### 3.6. Fourier Transform Infrared Spectroscopy (FTIR)

FTIR spectra of almond oil capped ZnO NPs are shown in the Figure 6. A broad band at higher wavenumbers of ~3640–3100 cm^−1^ and at lower wavenumbers of 1590–1650 cm^−1^ is due to O-H stretching and bending, respectively. All other peaks are attributed to the characteristics of almond oil capped ZnO NPs. The band presence near at 1950–2200 cm^−1^ indicates the CO adsorption on the surface of the oxide. Around 1530–1300 cm^−1^, C$\dddot{-}$C and C=O stretching appears. Normally, two or three bands occur in this range, i.e., ∼1450 cm^−1^ and 1340 cm^−1^; these bands are typically assigned to rings C$\dddot{-}$C and C=C, respectively. The C-O vibration is normally observed at 1030 cm^−1^. An IR band observed at 914 cm^−1^ can be attributed to the phytoconstituents, including oleic acid [68], present in almond oil [69]. Likewise, the bands at 950–750 cm^−1^ pertain to the Zn^+2^ internal stretching with oxygen, i.e., the Zn–O bond. The band at ~800 cm^−1^ points to the formation of metal oxide. Specifically, the peak at 650 cm^−1^ is the characteristic absorption of the Zn–O bond observed at all molar ratios. For samples synthesized at molar ratio 0.5:1, a shoulder has also been observed along with this band. It was earlier reported that a coalescence or emergence of NPs would appear in the form of double peaks [70]. As the molar ratio increases to 1:1, this kink nearly disappears, which clearly explains the formation of well-defined NPs due to presence of almond oil [71]. These well-defined NPs were observed in SEM images.

### 3.7. UV-Vis Spectra

**Figure 7:**
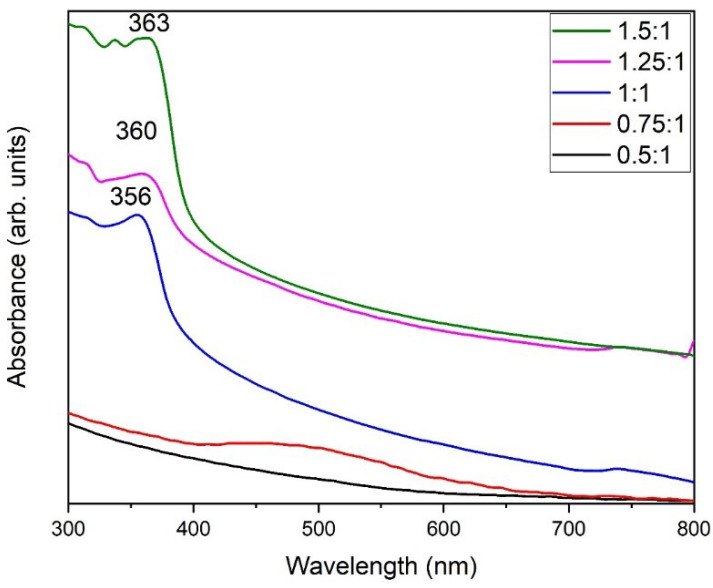
UV-Vis spectra of almond oil capped ZnO NPs prepared at different molar ratios.

ZnO exhibits optical properties that make it suitable for photocatalytic activity, as well as for other optical devices. Figure 7 represents the UV-Vis spectra of chemically synthesized NPs at different molar ratios. The ZnO spectra exhibit characteristically high absorption rates for higher solute concentrations or molar ratios, e.g., 1:1, 1.25:1, and 1.5:1. The wavelength of the maximum absorbance of almond oil stabilized ZnO NPs appears at 356 nm for molar ratio 1:1. The estimated band gap values of ZnO NPs was found to be 3.41–3.48 eVv. The decrease in the band gap due to a redshift towards a longer wavelength for the nanoparticles may be associated with various parameters, including the crystalline size, structural parameter, carrier concentration, the presence of a very small amount of impurities, and lattice strains [72,73]. For the ZnO nanoparticles, the suitable band gap for various applications is estimated to be between 3.0 and 3.5 eV at room temperature [74]. The absorption spectra of the ZnO NPs has been affected by the particle size; larger particles show enlarged scattering, which produces a redshift resulting in a decrease in band gap value. The concentration of NaOH plays a vital role in altering the size of the ZnO NPs. At different pH values, the number of *OH^−^* ions altering the nucleation rate and subsequently, the particle size, varies, a result that is in good agreement with those in the literature [13,75], and which is also confirmed by XRD (Figure 2) and SEM (Figure 4).

### 3.8. Photocatalytic Activity

Typically, the size and shape of the nanoparticles plays an important role in determining the photocatalytic properties of the nanomaterials, based photocatalysts. In this case, ZnO NPs prepared by using equimolar ratios of the precursor and the base (1:1), which delivered relatively small sized NPs (30 to 40 nm) at pH 9, were used as photocatalysts. This sample was selected, as the smaller sized NPs exhibit a larger surface area, which is crucial for photocatalytic activity. ZnO NPs (pH 9) were used as photocatalysts for the photocatalytic degradation of methyl orange dye under UV light irradiation. For this purpose, different concentrations of dye were used, including 25 mg/L, 50 mg/L, and 75 mg/L, and different amounts of photocatalyst were employed, including 0.3 g/L, 0.6 g/L, and 0.9 g/L. 

#### 3.8.1. Blank Test

**Figure 8:**
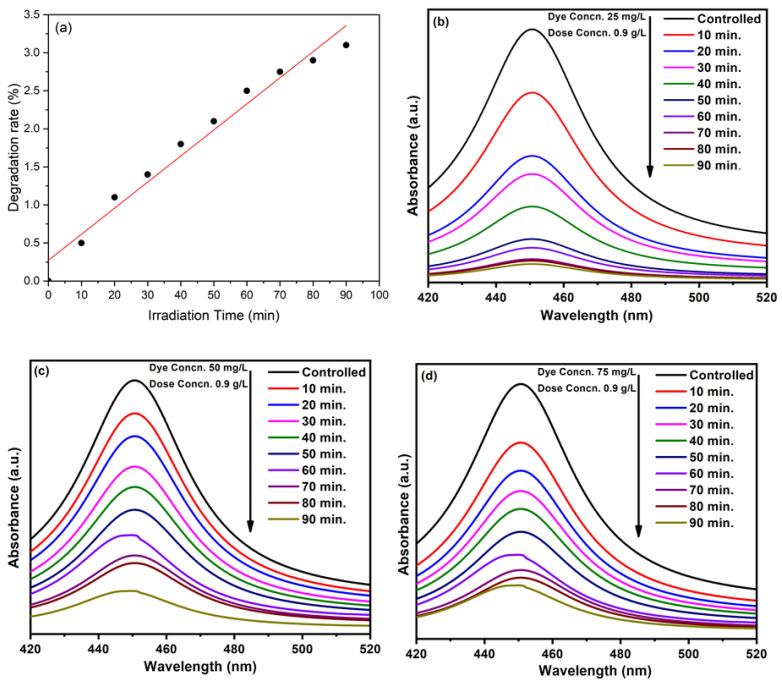
(**a**) Blank test for the degradation of MO without a catalyst. Photocatalytic activity of almond oil stabilized ZnO NPs against MO at different concentrations of dyes (**b**) 25 mg/L, (**c**) 50 mg/L, and (**d**) 75 mg/L.

Initially, a blank experiment was performed, without the addition of ZnO NPs (Figure 8a). A 25 mg/L dye solution was used, which was irradiated under UV. In the absence of a photocatalyst, only a 2.5% degradation was observed after 60 min of irradiation, which confirms the importance of a photocatalyst. 

#### 3.8.2. Dye Concentration:

**Figure 9:**
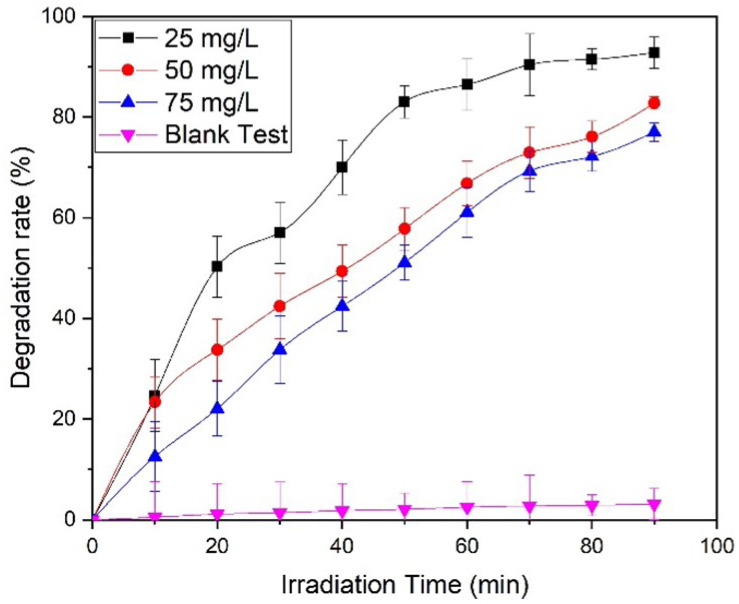
Degradation rate of MO for different dye concentrations and in comparison with a blank test.

Almond oil capped ZnO NPs (0.9 g/L) synthesized at molar ratio 1:1 (pH 9) were dissolved in 25 mg/L dye using 50 mg/L and 75 mg/L of solutions and irradiated for 90 min. The maximum degradation was achieved in the case of the lowest concentration of the dye, i.e., at 25 mg/L dye, which delivered a degradation of ~93% of the dye in 90 min (Figure 8b), whereas MO dye delivered in 50 mg/L and 75 mg/L solutions exhibited degradations of 83% and 79%, respectively (Figure 8b–d). The results revealed that the photodegradation rate increases with a decrease in dye concentration. Therefore, the optimum dye concentration is used to obtain a higher degradation rate. The lower photocatalytic activity at high concentrations of the dye can be attributed to the fact that a large number of dye molecules typically adsorbed on the surface of the photocatalyst and undesirably covered the active site of the photocatalyst. Moreover, the dye molecules also interfered with the UV light and the formation of radicals; thus, the activity of the photocatalysts decreased [76]. The degradation rate obtained here is comparatively higher than those presented in the literature [77,78]. Figure 9 represents the dye degradation rate for different dye concentrations. 

#### 3.8.3. Concentration Effect of ZnO NPs Photocatalysts

**Figure 10:**
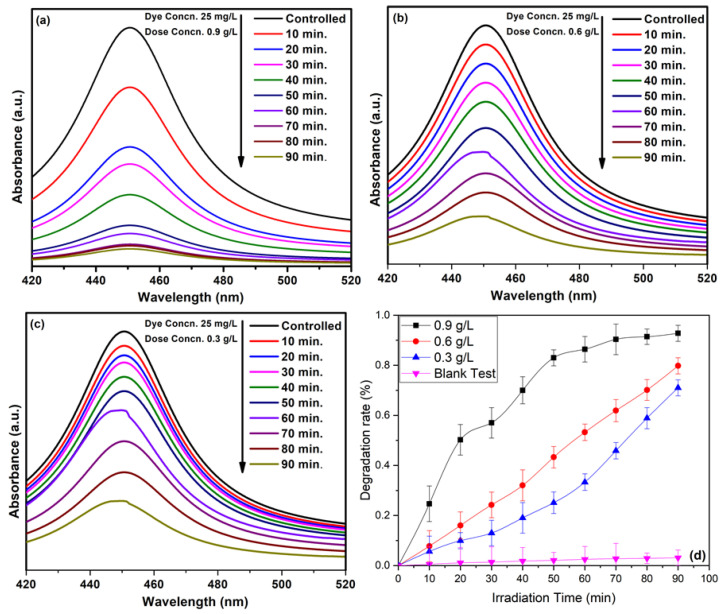
Photocatalytic activity at ZnO dosages (**a**) 0.9 g/L, (**b**) 0.6 g/L, and (**c**) 0.3 g/L; (**d**) degradation rate of MO for various ZnO dosages.

The effect of different dosages of ZnO was also studied, and the results are presented in Figure 10. For this purpose, the 25 mg/L concentration of MO solution was selected, in which 0.3 g/L, 0.6 g/L, and 0.9 g/L ZnO NPs were dispersed. Figure 10 shows that the mass of the photocatalytic material enhances the efficiency of MO degradation. As the dosage of ZnO increases, a large surface becomes available for the adsorption of the reactant molecules, providing a greater number of active sites [79]. Here, a nrealy 93% degradation was achieved by using the optimum dosage of ZnO (0.9 g/L). A comparison of the degradation rates at different dosages of ZnO is presented in Figure 10b. Table 2 shows the comparative study of the photocatalytic activity of ZnO with the results in literature, both in the absence and presence of a dopant/surfactant. 

#### 3.8.4. Kinetic study

**Figure 11:**
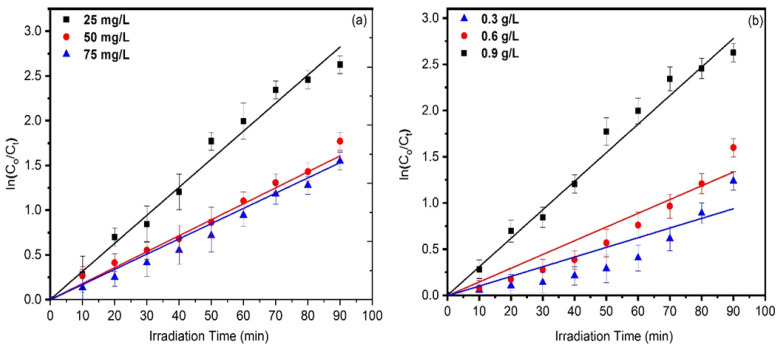
Kinetic study of almond oil capped ZnO NPs for different (**a**) Dye concentration and (**b**) ZnO dosages.

To study the kinetic mechanism of solvothermally synthesized almond oil capped ZnO, Equation (8) [80] was used, and it is presented in Figure 11a,b.

(8) $$ln\frac{C_{o}}{C}=kt$$

where *C_o_* = initial conc. of MO, *C* = conc. of dye after irradiation, *t* = time, and *k* = pseudo first order rate constant.

Figure 11a,b represents the linear plot of $ln\frac{C_{o}}{C}$ vs. the exposure time for both variations. It is observed that the pseudo first order rate constant was followed. Different extracted parameters from the graphs are presented in Table 2. The values of R^2^ > 0.92942 and K ~ 0.04581 min*^−^*^1^ confirm the good fit of the model and the rapid degradation of MO.

#### 3.8.5. Degradation Mechanism

**Figure 12:**
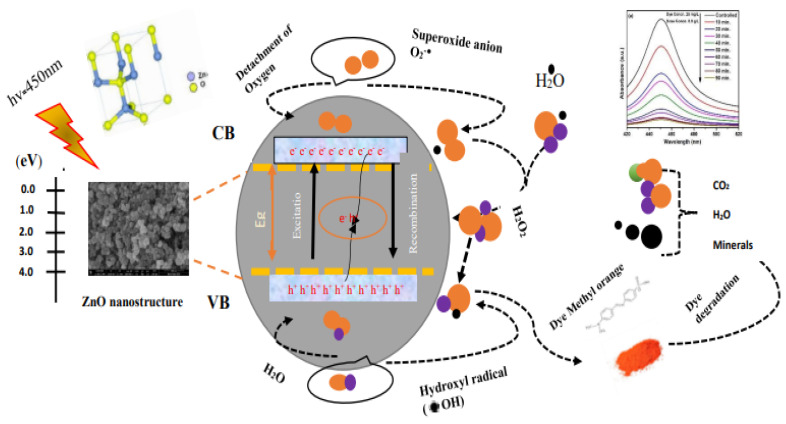
Schematic representation of degradation mechanism of ZnO NPs against MO.

Figure 12 and Equations (9)–(22) [12,81,82,83,84,85,86] present the degradation mechanism of MO. When nano-sized particles react with dye, the oxidation and reduction phenomena occur. Light absorbed by these nano-sized particles facilitates the breaking down of the dye molecules by the formation of various ions, etc. The main species formed in the photodegradation mechanism are hydroxyl radicals and superoxide anions [84]. According to Rakhshaei et al., ZnO NPs exhibit high photocatalytic capabilities [85]. UV irradiation facilitates the formation of electron hole pairs and the subsequent excitation of the electrons towards the conduction band (CB). Light triggered most of the charged particles to undergo a recombination process; on the other hand, several other particles underwent the oxidation reduction reactions. These light-triggered particles facilitate the generation of superoxide anions (•O^2−^), hydrogen peroxide fragments (H_2_O_2_), hydroxyl radicals (•OH), hydrogen dioxide anion (HO^2−^), and (•HO_2_) [12,86]. As discussed previously, the most reactive species, such as •OH and O_2_^-^, may be responsible for the degradation of dye. A study has shown that reactive oxygen species (ROS), such as •OH, are mainly responsible for dye degradation. With increased ROS generation, the degradation process also increases, which helps to remove pollutants.

AO + hv → AO (e + h)(9)

Here, A = photocatalyst

AO (e^−^) + O_2_ → AO + ^•^O_2_^−^ (10)

AO (e^−^) + ^•^O_2_^−^ +2H^+^ → AO + H_2_O_2_ (11)

AO (e^−^) + H_2_O_2_ → MO + ^•^OH + OH^−^ (12)

^•^O_2_^−^ + H_2_O_2_ → ^•^OH + OH^−^ + O_2_ (13)

^•^O_2_^−^ + H^+^ → ^•^OH_2_ (14)

AO (e^−^) + ^•^OH_2_ → AO + HO_2_^−^ (15)

HO_2_^−^ + H^+^ → H_2_O_2,_ (16)

2^•^OH_2_ → O_2_ + H_2_O_2_ (17)

Now, the redox mechanisms initiated with light-triggered holes are

AO (h^+^) + H_2_O → AO + ^•^OH + H^+^ (18)

AO (h^+^) + H_2_O → AO + ^•^OH + H^+^ (19)

AO (h^+^) + OH^−^ → AO + ^•^OH (20)

Consequently,

^•^OH + H^+^ + 2e^−^ → H_2_O (21)

1/2O_2_ + 2H^+^ + 2e^−^ → H_2_O (22)

#### 3.8.6. Reproducibility and Stability 

**Figure 13:**
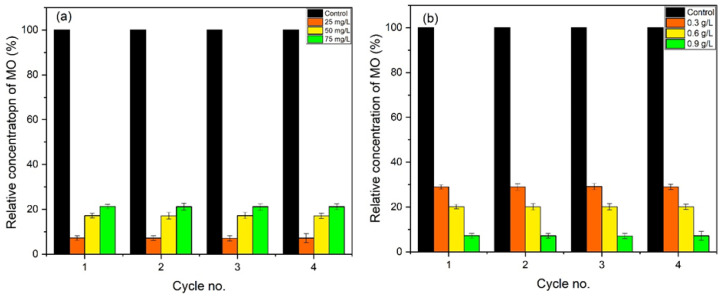
Reproducibility and stability of almond oil capped ZnO NPs against MO for various (**a**) dye concentrations and (**b**) ZnO dosages.

The stability and reproducibility of almond oil capped ZnO NPs were also examined for up to 3 cycles. Figure 13a,b represents the degradation process; in this case, the catalyst remained stable up to 3 cycles. No significant difference in values was observed, confirming that these synthesized almond oil capped ZnO NPs can be effectively re-used for several cycles. 

### 3.9. Antibacterial Activity

**Figure 14:**
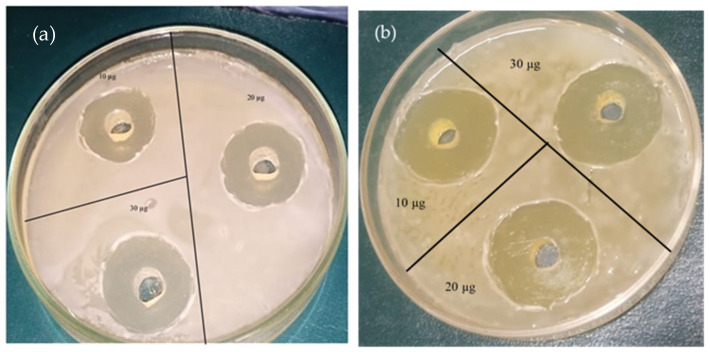
Antibacterial activity of ZnO NPs against (**a**) E. coli and (**b**) Pseudomonas aeruginosa.

Apart from the photocatalytic activity, the antimicrobial potential of the as-prepared almond oil capped ZnO NPs was also highlighted. For this purpose, *E. coli* and Pseudomonas aeruginosa bacteria strains were selected, and the results are displayed in Figure 14a,b. These synthesized NPs showed a stronger antibacterial effect, inhibiting the growth of *E. coli* up to 29 mm (Figure 14a) and up to 32 mm in *Pseudomonas aeruginosa* (cf. Figure 14b). Almond oil contains vitamin A, oleic acid, etc., substances which are known to exhibit strong antibacterial activity. Typically, ZnO NPs may exert their antimicrobial activity in various ways, but the main potential mechanism of action is described below, although there may be more than one mechanism responsible for the antimicrobial activity of the NPs, which makes the determination of the main mechanism more difficult. Generally, in aqueous suspensions, ZnO NPs demonstrate chemical interactions between hydrogen peroxide and the proteins of the cell membrane. This prompts the formation of the specific species, which may explain the special antimicrobial activities of the NPs [87]. The proposed mechanisms are (i) the production of reactive oxygen species (ROS) [88], (ii) the loss of cell integrity following contact between ZnO NPs and the cellular wall [89], (iii) the discharge of Zn^2+^ ions [90], and (iv) ZnO NPs internalization [91]. ROS formation via metal oxide NPs is one of the common and accountable mechanisms for antimicrobial action in the literature. ROS consist of superoxide anions (O_2_^−^), hydroxyl radicals (HO^−^_2_), and hydrogen peroxide (H_2_O_2_), which may cause the destruction of cell components, as well as proteins, DNA, and lipids [92].

The qualitative antimicrobial properties were analyzed using the disk diffusion method, in accordance with Clinical Laboratory Standard Institute standards (CLSI 2021, Annapolis Junction, MD, USA). For the measurement of the inhibitory efficiency of the samples, the microbial inocula from 18 h nutrient agar was developed by using the 0.5 McFarland standard. Petri dishes containing Mueller–Hinton agar (MHA) were inculcated with the prepared microbial inocula. Solutions containing 10, 20, and 30 µg of the sample synthesized with molar ratio 1.5:1 were stained on 6 mm sterilized paper discs, previously arranged on the surface of the medium. For proper diffusion of the compounds, the dishes were kept at room temperature, followed by a 24 h incubation at 37 °C. The antimicrobial activity was calculated by determining the inhibition zones diameters [93]. Ciprofloxacin was used as the control. 

A broth microdilution method was used to test the MIC (minimum inhibitory concentration), according to CLSI procedure. The binary dilution of every compound was carried out in the broth media, with the calculated concentration for ZnO NPs within a range of 20 µg/mL to 0.0390625 µg/mL. In addition, 10 μL of microbial inocula, adjusted at 1.5 × 10^7^ CFU/mL, was added [94]. After 24 h of contact with this sample, the viable count showed a reduction of 94 ± 3% in the liquid broth. The inhibition zones of both Gram-positive and Gram-negative bacteria, along with statistical analysis, are presented in Table 3.

### 3.10. Antioxidant Study 

**Figure 15:**
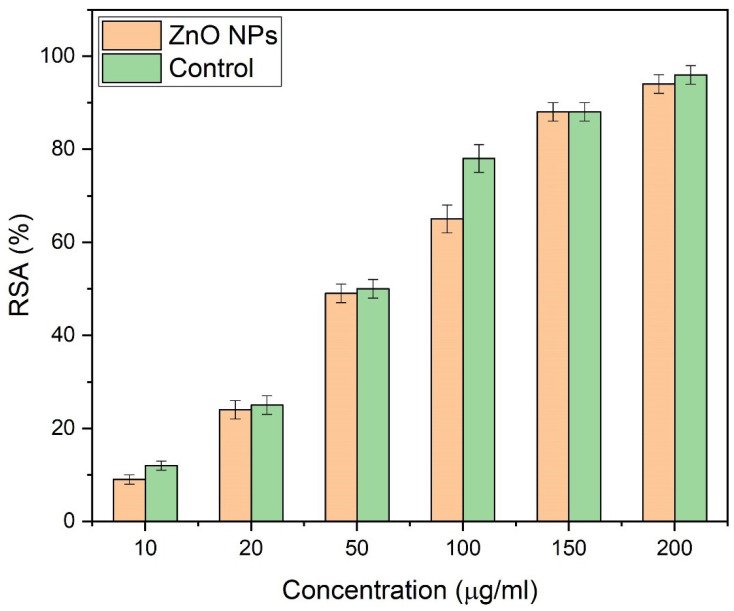
Free radical scavenging activity of ZnO NPs.

Almond oil capped ZnO NPs also showed good antioxidant properties. Important phytoconstituents, such as vitamin A, oleic acid, etc., present in almond oil acted as significant antioxidants. The activity was studied by using 10, 20, 50, 100, 150, and 200 µg/mL of ZnO NPs. The free radical scavenging activity (RSA) enhances with the increased concentrations of ZnO, as presented in Figure 15. Hydrothermally synthesized ZnO NPs exhibited excellent (94%) antioxidant properties at 200 µg/mL, which is higher than the rates reported in the literature [95]. Furthermore, the introduction of NaOH (as an ordinary resistance) may trigger the generation of reactive oxygen species (ROS), which leads to the enhancement of the antioxidant properties of the NPs. ROS, such as O_2_^−^, OH^-^, and H_2_O_2_, are enhanced by drought stress. Statistical analysis of antioxidant activity is provided in Table 4; ascorbic acid was used as the control.

## 4. Conclusions

Spherical-shaped, almond oil capped ZnO NPs were synthesized by an eco-friendly solvothermal method. In this study, the use of almond oil prevented the agglomeration and enhanced the efficiency of as prepared ZnO, which is utilized for different applications. The NPs were prepared by using different molar ratios of Zn(CH_3_COO)_2_.6H_2_O:NaOH, 0.5:1, 0.75:1, 1:1, 1.25:1, and 1.5:1, which yielded different pH values, 11. 10, 9, 8, and 7, respectively. XRD results exposed a hexagonal structure, with high crystallinity, for all samples. Gaussian analysis revealed that the FWHM of the samples increased with an increase in molar ratios for all samples, except for that reflecting the 1.5:1 molar ratio. The decrease at pH 7 was due to the presence of more solute atoms, leading to a coalescence of the particles. SEM images showed that samples synthesized at molar ratio 0.5:1 (pH = 11) consisted of relatively dense and larger NPs. However, the other samples obtained at an equivalent molar ratio of 1:1 (pH = 9) demonstrated the preparation of well-dispersed, spherical shaped, relatively smaller sized ZnO (30–40 nm), due to the presence of fewer *OH*^−^ ions, which controlled the nucleation and growth of the NPs. In all the samples, the presence of almond oil as a capping agent led to the formation of well-dispersed and stable NPs. The photocatalytic activity of the sample with molar ratio 1.5:1 was tested regarding the degradation of MO, and it demonstrated an efficient degradation of the dye (~93%), with a relatively higher rate constant. Almond oil capped ZnO NPs also exhibited high antibacterial activity (~94% RSA) due to the generation of ROS, which effectively inhibited the growth of *E. coli* bacteria. 

## Figures and Tables

**Table 1:** Calculation of different lattice parameters.

Molar Ratio	a (Å)	c (Å)
0.5:1	3.27	5.69
0.75:1	3.27	5.64
1:1	3.26	5.63
1.25:1	3.26	5.64
1.5:1	3.36	5.65

**Table 2:** Comparison of ZnO photocatalytic activity with results from the literature.

Catalyst	Surfactant	Dopant	Dye Concentration (ppm)	Degradation Time (min)	Photo Degradation (%)	References
ZnO	Pithecellobium dulce peel		-	50	~63	[39]
ZnO		Ag-r-GO	20	120	95.0	[40]
ZnO			10	50	80.8	[41]
ZnO		Urea	10	30	92	[42]
ZnO	-		25	90	93	Present Study

**Table 3:** Antibacterial activity of the almond oil capped ZnO NPs and statistical analysis.

Microorganism	1.5:1		Ciprofloxacin	
	IZ ^1^	MIC ^2^	IZ ^1^	MIC ^2^
*E. coli*	29.0 ± 1.0^3^	0.30 ± 0.01^1^	28.1 ± 1.0^3^	6.0 × 10^−3^
*Pseudomonas aeruginosa*	32.1 ± 1.1^3^	0.26 ± 0.01^1^	33.0 ± 1.6^3^	8.0 × 10^−3^

^1^ IZ, diameter of inhibition zones (mm), including disc diameter of 6 mm. ^2^ MIC, minimum inhibitory concentration (mg ML^−1^). ^3^ Values are mean ± standard deviation of three different samples, analyzed individually in triplicate.

**Table 4:** Statistical analysis of RSA of ZnO NPs.

Concentration (µg/mL)	ZnO NPs (Cycle 1) ^4^	ZnO NPs (Cycle 2) ^5^	ZnO NPs (Cycle 3) ^6^	Control	Mean ^3^	Standard Deviation ^3^
10	9	9.1	9.23	12	9.11	0.09
20	24	24.2	24.12	25	24.11	0.08
50	49	49.1	48.59	50	48.90	0.22
100	65	65.1	65.1	78	65.07	0.05
150	88	87.89	87.88	88	87.92	0.05
200	94	94.1	93.73	96	93.94	0.16

^3^ Values are mean ± standard deviation of three different samples, analyzed individually in triplicate. ^4^ Antioxidant activity of ZnO NPs against DPPH in first replicate. ^5^ Antioxidant activity of ZnO NPs against DPPH in second replicate. ^6^ Antioxidant activity of ZnO NPs against DPPH in third replicate.

## Data Availability

Data are contained within the article and the Supplementary File.

## Supplementary Materials

The following supporting information can be downloaded at: https://www.mdpi.com/article/10.3390/ma16145011/s1, Figure S1: Corresponding XRD fitting diagram of hydrothermally synthesized almond oil ZnO at different molar ratios (**a**) 0.5:1, (**b**) 0.75:1, (**c**) 1:1, (**d**) 1.25:1 and (**e**) 1.5:1.
