# Supplementary material for: Evaluation of Photocatalytic, Antioxidant, and Antibacterial Efficacy of Almond Oil Capped Zinc Oxide Nanoparticles
Source: Materials (Basel). 2023 Jul 15;16(14):5011. doi: 10.3390/ma16145011 (PMC10381886; doi:10.3390/ma16145011)
Supplement: Supplementary file 1 [file materials-16-05011-s001.zip › materials-2447555-supplementary.pdf]

materials

Supplementary File

Evaluation of Photocatalytic, Antioxidant, and Antibacterial Efficacy of Almond Oil Capped Zinc Oxide Nanoparticles

Iqra Ramzan [1], Mahwish Bashir [1], Adnan Saeed [1], Babar Shahzad Khan [1,*], Mohammed Rafi Shaik [2], Merajuddin Khan [2], Baji Shaik [3] and Mujeeb Khan [2,*]

[1] Department of Physics, Government College Women University, Sialkot 51310, Pakistan; ramzaniqra9@gmail.com (I.R.); mahwish.bashir@gcwus.edu.pk (M.B.); adnan.saeed@gcwus.edu.pk (A.S.)
[2] Department of Chemistry, College of Science, King Saud University, P.O. Box. 2455, Riyadh 11451, Saudi Arabia; mrshaik@ksu.edu.sa (M.R.S.); mkhan3@ksu.edu.sa (M.K.)
[3] School of Chemical Engineering, Yeungnam University, Gyeongsan 38541, Republic of Korea; shaikbaji@yu.ac.kr
* Correspondence: babar.shahzad.khan@gcwus.edu.pk (B.S.K.); kmujeeb@ksu.edu.sa (M.K.); Tel.: +966-1146-70439 (M.K.)

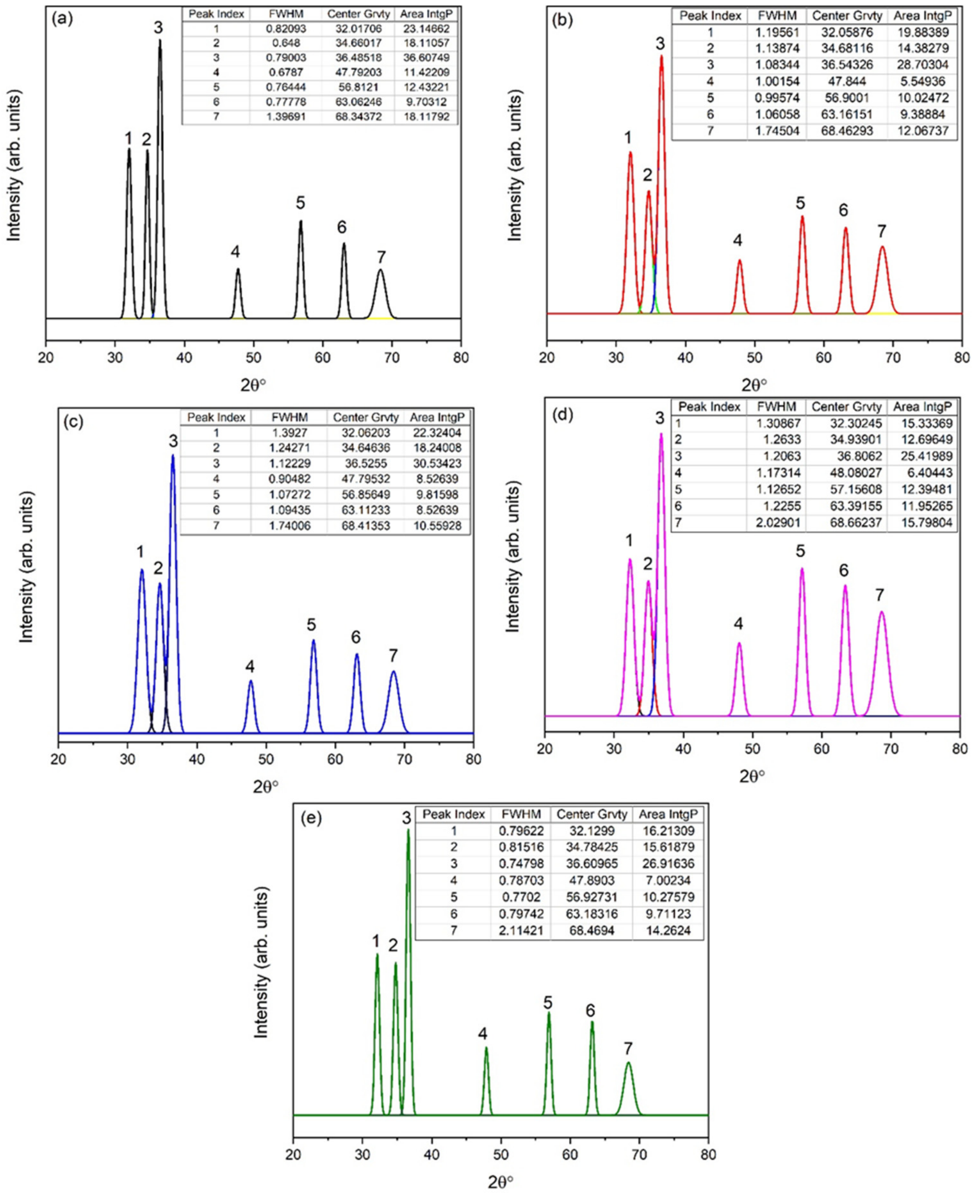

Figure S1: Corresponding XRD fitting diagram of hydrothermally synthesized almond oil ZnO at different molar ratios (**a**) 0.5:1, (**b**) 0.75:1, (**c**) 1:1, (**d**) 1.25:1 and (e) 1.5:1.
